# Mechanism of DNA Chemical Denaturation

**DOI:** 10.1021/acsomega.5c05005

**Published:** 2025-10-01

**Authors:** Daniel A. Ostrovsky, Mikhail V. Ostrovsky

**Affiliations:** Independent Researcher, Los Angeles, California 90024, United States

## Abstract

We developed a method to evaluate the degree of influence of attraction and electrostatic repulsion forces in DNA during its chemical denaturation. Our approach shows that when a solution can split apart a target molecule, the forces inside the molecule can be deduced by analyzing the properties of the surrounding solution. Our method is suitable for selecting DNA (or other systems with controllable denaturation) targeted for specific applications or to optimize the denaturants for any given DNA. Our theory has been developed for the chemical denaturation of DNA for low- and medium-denaturation degrees, including the denaturation of 50% as a reversible first-order reaction. Specifically, we show the degrees of influence of hydrogen bonding, dispersion, polar forces, proton donor/acceptor ratio, dipole induction, orientation parameter, and electrostatic interaction on the denaturation process of DNA. The absolute enthalpy values for DNA chemical denaturation are significantly lower than those in the thermal denaturation process (where values are positive). We show that the mechanism for reaching 50% DNA denaturation differs thermally and chemically. The thermal denaturation process mainly involves breaking hydrogen bonds via heating, while the chemical denaturation process involves replacing DNA’s hydrogen bonds with denaturants. We show that hydrogen bonding is the dominant enthalpic contributor to the chemical denaturation of T4 bacteriophage DNA, and the proton-donor effect is the dominant mechanism for disrupting hydrogen bonds during DNA denaturation. The influence of this effect is two times greater than that of the proton-acceptor effect. We also show that the orientation component is another essential factor for DNA denaturation, which is part of the polar cohesion parameter. We show that the total cohesion parameter measured at 50% of DNA chemical denaturation represents the electrostatic (repulsion) forces that maintain the DNA helix. The conclusions above were achieved using the cohesive energy density approach and corresponding equations based on the thermodynamics of the denaturation process. Independent experimental data, which we analyzed using our theory, support these conclusions.

## Graphical abstract

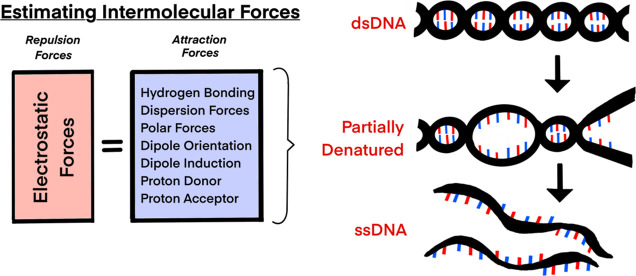

## 1 Motivation and Objectives

### 1.1 Motivation and Justification of the Work

DNA denaturation plays a central role in many biological processes. It is therefore important to study the forces that maintain the integrity of the double-stranded DNA helical structure because of its central role in biotechnology and medicine. Analyzing DNA denaturation is vital to understanding fundamental genetic processes such as replication and transcription.

Other applications of DNA denaturation are bioanalytical methods and molecular diagnostics. One such method is PCR (polymerase chain reaction), where controlled denaturation/renaturation of DNA is a critical step. DNA denaturation plays a central role in applications such as gel electrophoresis, gel electrophoresis by denaturation gradient, and denaturation sequencing.^1^ There are applications where wholly or partially denatured DNA is used, such as nanotechnology,^2–4^ the creation of nanodevices,^5^ new or improved medical applications such as new drug delivery systems,^6^ study drug–DNA interactions,^7^ analyze forensic evidence,^8^ design molecular memories,^9^ and build functional DNA sensors that include metal detection, where this process involves denaturation and subsequent renaturation steps.^10^

Thus, we are motivated to study DNA denaturation because of its essential role in improving our knowledge of biological processes, advancing bioanalytical techniques, and improving molecular diagnostic methods, as well as in other medical applications, forensic science, DNA-based sensors, and nanodevice-related applications.

A significant number of works have been devoted to thermal denaturation, while chemical denaturation has been insufficiently studied and therefore requires further investigation, as discussed in the literature review.

### 1.2 Purpose of the Work (Objectives) and Our Approach

The main objective of our work is to develop a method that quantitatively reveals the role of different intermolecular forces in DNA that are responsible for maintaining its helical structure. We use controlled chemical denaturation of DNA as a tool to detect and evaluate these forces. The theory of this denaturation process and the corresponding mathematical protocol should be developed. Our approach shows that when DNA can be split apart by a solution in which it is placed via denaturation, the forces inside the molecule can be deduced by analyzing the properties of the surrounding solution. This is attractive since it will enable the calculation of inaccessible properties of DNA (or other polyelectrolytes) from knowledge of known or easily measurable properties of the surrounding solution, which are well-understood.

## 2 Technical Overview

Our objective is to analyze the forces that maintain the integrity of the double-stranded DNA (dsDNA) molecule. Our main result is to prove that the cohesion parameters of dsDNA at its melting temperature (50% denaturation) are equal to the cohesion parameters of the surrounding solution.

Our analysis is based on the laws of thermodynamics that allow us to reveal the forces that act *within* dsDNA using properties of the surrounding solution.

We first give an overview of our technical approach that explains the main ideas before a more in-depth presentation in Section 4.

The denaturation process can be described as a consecutive transition from double-stranded (DS) to partially denatured (PD) and then to two single-stranded (2SS) parts. The first step of this process is a first-order process described by the equilibrium constant *K*

1 $$K=\frac{\left[PD\right]}{\left[DS\right]}$$

The second step of this process is a second-order process described by the equilibrium constant *K*′

2 $$K^{\prime }=\frac{{\left[SS\right]}^{2}}{\left[PD\right]}$$

We analyze the first step of the denaturation process above, which consists of at least 50% denaturation.

According to van’t Hoff,^11^ $\ln K=A-\frac{\Delta H}{RT}$ , where *A* is a constant, Δ*H* is the denaturation enthalpy, *R* is the universal gas constant, and *T* is the absolute temperature. When DNA has reached 50% denaturation or the equilibrium constant *K* = 1, we have the constant term $A={\left(\frac{\Delta H}{RT}\right)}_{T_{m}}$ and the Van’t Hoff equation has the following form

3 $$\ln K={\left(\frac{\Delta H_{DNA}}{RT}\right)}_{T=T_{m}}-\frac{\Delta H}{RT}$$

where the subscript DNA refers to the properties of DNA. Hildebrand^12,13^ developed the following equation to describe the enthalpy of an exothermic process.

4 $$-\Delta H_{1}=\delta^{2}V+RT$$

where δ^2^ is the cohesion energy density (energy per volume) and *V* is the molar volume of a chemical. We applied this equation to chemical denaturation.

Substitution of the Δ*H* from eq 4 into [Disp-formula eq3] gives

5 $$\ln K=\frac{{\left(\delta^{2}V\right)}_{sol}}{RT}-{\left(\frac{{\left(\delta^{2}V\right)}_{DNA}}{RT}\right)}_{T_{m}}$$

where the subscript sol refers to the properties of the solution surrounding DNA. At 50% denaturation, ln *K* = 0, and eq 5 becomes

6 $${\left(\delta^{2}V\right)}_{DNA,T_{m}}={\left(\delta^{2}V\right)}_{sol,T_{m}}$$

This means that the properties in the DNA are equal to the properties of the solution at the melting temperature, allowing us to uncover the intermolecular forces what is in the DNA using chemical denaturation. When we use the solubility parameters, they are only applicable to small, uncharged molecules. By knowing that at the equilibrium point of 50% denaturation, the properties of the solution are identical to the properties of DNA, we can apply these measurable solubility parameters in the solution to deduce forces inside DNA.

Hansen^14,15^ and Karger, Keller, Snyder, and Eon^16–19^ split up the δ^2^ total cohesion parameter into 3 and 5 fractional cohesion parameters, respectively

7 $$\delta^{2}=\delta_{d}^{2}+\delta_{h}^{2}+\delta_{p}^{2}$$

8 $$\delta^{2}=\delta_{d}^{2}+\delta_{o}^{2}+2\delta_{i}\delta_{d}+2\delta_{a}\delta_{b}$$

Hansen split the total δ^2^ into three fractional cohesion parameters related to the following intermolecular forces: dispersion forces (δ_d_), hydrogen bonding (δ_h_), and polar forces (δ_p_). Karger, Keller, Snyder, and Eon split it into 5: dispersion forces (δ_d_), dipole orientation (δ_o_), dipole induction (δ_i_), hydrogen bonding related to proton donors (δ_a_), and hydrogen bonding related to proton acceptors (δ_b_).

Equation 4 has been developed for a single-component system for associated solutions with no or only positive deviations from Raoult’s law.^20^ We expanded this equation to multicomponent systems, assuming the additivity principle, which states that the influence of each component in the system is proportional to its volume fraction in terms of enthalpic contribution

9 $$-\Delta H=\left(\sum_{i=1}^{i=j}\delta_{i}^{2}V_{i}V_{f_{i}}\right)+RT$$

*V*_f_*i*__ is the volume fraction of a component, *i*, in the system with *j* components, and each fractional cohesion parameter and molar volume of each component is calculated as a pure substance.

For multi component systems eqs 5 and 6 have the form

10 $$\ln K={\left(\frac{\sum_{i=1}^{i=j}\delta_{i}^{2}V_{i}V_{f_{i}}}{RT}\right)}_{sol}-{\left(\frac{{\left(\delta^{2}VV_{f}\right)}_{DNA}}{RT}\right)}_{T_{m}}$$

11 $${\left(\delta^{2}VV_{f}\right)}_{DNA,T_{m}}={\left(\sum_{i=1}^{i=j}\delta_{i}^{2}V_{i}V_{f_{i}}\right)}_{sol,T_{m}}$$

This allows us to measure the distribution of individual forces inside the DNA using the distribution of forces inside the solution at 50% denaturation. Additionally, the sum of all intermolecular attraction forces in eq 11 equals the electrostatic repulsion forces in DNA.

This theory has been supported in the experimental part of the paper and has been used to develop a method to quantitatively reveal the influence of different intermolecular forces in DNA that are responsible for maintaining its helical structure.

Alternative approach. An alternative approach to calculating the change in internal energy is possible, considering three consecutive steps during denaturation. Step 1: Breaking of base pairs/hydrogen bonds in the transition from dsDNA to 2ssDNA. (cohesive forces inside DNA) Step 2: Denaturant molecules are separated from the solution (Solution cohesion forces.) Step 3: A denaturant interacts with the ssDNA regions (Cohesive forces between DNA and denaturant). The energy values for each step can be expressed in terms of total (not fractional) cohesion parameters, i.e., revealing different acting forces presented in our approach above is impossible in the discussed alternative approach. If the change in internal energy (enthalpy) is split in these three steps, the solvent-related cohesive energies available can be used only for step 2 and partially for step 3. Thus, this approach does not give sufficient information to judge the forces that act in the denaturation process. In addition, step 1 in the alternative approach above considers only the breaking of one type of force inside DNA–hydrogen bonding. Other forces maintaining the integrity of helix structure (dispersion, polar, orientation effect) in this approach were neglected. That is why we did not consider this approach and applied the thermodynamic approach with a simple model and minimum limitations and assumptions.

Limitations of our approach. Current limitations of our approach include (a) limitations on which Hildebrand equation—with Hansen^14,15^ and Karger, Snyder, and Eon^17^ modifications—is based, and (b) on the additivity principle for components in the solution surrounding DNA included in our derivations. In other words, it does not apply to systems in which cosolvents and denaturants are ionized and in the presence of salt. However, the method allows one to make a judgment about a polyelectrolyte such as DNA. Experiments presented in this article show that these limitations are acceptable in the presence of small concentrations of salts contained in some buffers.

Predictive power of our approach and method. Our approach allows for the selection of denaturants with perfect denaturing power; expression 10 demonstrates that such denaturants should have a high value of the product δ_i_^2^ *V*_f_. At the maximum *V*_f_ value, the *V*_f_ term is the solubility of the denaturant. The selection of denaturants should consider the highest fractional δ^2^ for the given DNA. For example, the denaturant selected for the effective denaturation of the DNA of the T4 bacteriophage should have a high value of the partial cohesion parameter δ_h_ related to hydrogen bonds and a high solubility in water to create a large product value δ_h_^2^ *V*_f_.

The method has been developed to detect the most influential fractional enthalpic component acting in the denaturation process. This method can be used to select a polyelectrolyte (DNA type) for different applications and to judge the types and relative influence of forces acting on the selected DNA type during denaturation.

## 3 Introduction—Literature Review

A DNA duplex is a double-stranded helix generally maintained by hydrogen bonding between the two strands of DNA. It is defined by the nucleotide pairings A-T and C-G. The term denaturation for DNA means the loss of bonding between nucleic acid pairs due to the addition of a solvent(s) or an increase in temperature. Each factor leads to the separation of double-stranded helical nucleic acids into single-stranded coils.

The rate and degree of the denaturation process depend on the following interrelated parameters:^21,22^ temperature, structure and size of DNA, and the composition of surrounding media (solvents,^23,24^ buffers,^25^ pH, and salts^26–28^ ). Many theoretical and experimental works have been published to describe factors related to DNA denaturation. However, the precise mechanism of DNA denaturation is still poorly understood and has continued to attract the attention of researchers.^29^

### 3.1 Theoretical Works Related to Thermal Denaturation of DNA without Solvents

In the present article, we discuss and analyze thermal denaturation for the purpose of comparing it to the chemical denaturation process in order to show the differences between the mechanisms.

The first well-known theoretical work on DNA denaturation by heating (without solvents), called the Poland–Scheraga model,^30,31^ was discussed by C. Richard and A. Guttmann.^32^ According to the authors: “the question of the mechanisms applied to real DNA denaturation process which explain behavior as observed in melting curves is still far from being satisfactorily answered.” The efforts toward improvement and further development or simplification of this model continue.^33–35^

Thermal denaturation of DNA using statistical mechanics for interacting and noninteracting loops has been studied by Kafri, Mukamel, and Peliti.^36^ In their paper, the Poland–Scheraga model of DNA denaturation is extended to analyze loop formation and their interaction within the molecule. This analysis was combined with the scaling theory for polymer networks. The authors concluded that the model exhibits critical behavior in some of its properties, such as the loop size distribution and the length of the segment. The model was extended to study the unzipping transition (denaturation) induced by an external force.

An additional study of external forces has been studied by Marenduzzo et al.^37^ Analyzing the dynamical scaling of DNA denaturation (unzipping transition), the authors theoretically compared DNA force-induced and thermal denaturation. The denaturation/unzipping dynamics on the phase boundary in the presence of a force are distinctly different from the thermal denaturation at zero force.

Another direction of theoretical work rapidly developing to understand the denaturation process is the Nearest-Neighbor method, where the DNA helix is treated by the interaction between neighboring DNA/DNA base pairs.^38^ Tinoco, Uhlenbeck, Levine^39^ calculated the thermodynamic parameters for the nearest neighbor sequence in DNA, SantaLucia, Hicks,^38,40^ and Sugimoto et al.^41^ Sugimoto presented results of measurements of the thermodynamic parameters of the DNA/DNA nearest-neighbor of 50 DNA/DNA duplexes. SantaLucia and Hicks^40^ developed a set of thermodynamic parameters describing the secondary structure of DNA. The authors^40^ presented a database of thermodynamic parameters for nearest neighbor base pairs needed to create programming algorithms to predict secondary DNA structures such as hairpins, internal loops, and mismatches. Based on this knowledge, SantaLucia created DNA software.

Using the nearest neighbor method, Dragan, Crane-Robinson, and Privalov^42^ conclude that hydrogen bonds between DNA base pairs have a completely entropic nature and these bonds are responsible for 40% of Gibbs free energy. van der Waals forces of enthalpic origin provide the remaining 60% of Gibbs free energy.

In contrast with the nearest-neighbor approach, Wartell and Benight^43^ created a theoretical model for DNA’s thermal denaturation (helix–coil transition) and compared the theory with experimental results. The comparison of theory with experiment (melting curves for short-segment DNAs) indicates that the base pair sequence has a relatively small influence on the stacking free energy. The agreement between theory and experiment is obtained for equilibrium transitions of 14 of 15 fragments 80–587 bp (base pairs) long. The deviation between theory and experiment for 516 bp DNA can be attributed to the formation of stem-loop structures. The explanation of inconsistent results observed with long DNA fragments was also attributed to the possible formation of loops.

Additionally, several models have been presented to predict the melting temperature of DNA *T*_m_ and to evaluate the influence of the factors describing *T*_m_ such as the length of DNA,^21,44,45^ (*T*_m_ decreases for shorter pieces), sequence of nucleotide composition,^46–48^ addition of salts (ionic strength, pH),^47–49^ and influence of solvents.

### 3.2 Theoretical Works Related to Thermal Denaturation of DNA in Solvent(s) Presence

A better understanding of the mechanism of thermal denaturation of DNA in the presence of solvent(s) has attracted the attention of many scientists.

Sinanoglu and Abdulnur^50^ show that the DNA double helix is stable in water but becomes denatured in some solvents. To reveal the role of water in keeping the helix together, the solvent contributions to the free energy difference between single strands and the helix are calculated for water, methyl alcohol, glycol, formamide, glycerol, ethanol, *n*-propanol, and *n*-butanol. The property of the solvent crucial for denaturation is found to be mainly the enthalpy part of the surface tension. In terms of entropy contribution to the Gibbs free energy, the authors concluded that base (DNA) dipole orientation affects the solvent dipoles. They also found that this effect depends on the change in dielectric constant with temperature.

Later Macedo, Guedes and Albuquerque^51^ studied the effects of solvent interaction on the nonlinear dynamical structure of a DNA segment using a time-independent perturbation approach. The authors investigated the denaturation temperature profiles and found that the melting temperature of DNA decreases as the solvent potential increases.

Another approach in the study of base stacking driving forces in DNA has been done by Chi H. Mak.^52^ He conducted large-scale molecular simulations to elucidate the thermodynamic parameters (driving forces) underlying the stacking interaction in DNA. To calculate the stacking free energy or Gibbs free energy, the author used the Monte Carlo calculation method and simulated purine and pyrimidine bases surrounded by many solvent molecules within a cubic box of up to 134 cubic angstroms in size. The author studied thermodynamic driving forces and the physicochemical origins behind DNA-solvent stacking interactions. The computer simulation leads the author^52^ to conclude that the entropy of the hydrophilic origin of the solvent is the major driving force for base stacking. At the same time, DNA backbone conformational entropy leads to destabilization of base stacking. These two opposing entropic effects almost compensate for each other, resulting in a mild total stacking-free energy of around 1 kcal·mol^–1^. Another conclusion of the author^52^ is that hydrogen bonding, charge–charge interaction, and dispersive forces have a small influence on DNA stability.

### 3.3 Experimental Works

In the last 50 years, a significant number of experimental works have been performed^24,53–57^ studying the role of chemicals (solvents) in DNA melting.

The articles in this section are subdivided into the following subgroups:

1. DNA thermal denaturation without solvents,
2. DNA denaturation by solvents at constant temperature.
3. Thermal denaturation in the presence of solvents

The second group we will call “chemical denaturation”, and the third group will be defined as thermal and chemical mixed denaturation. The third group includes experimental work studying the influence of solvents on DNA denaturation at different temperatures or, if the temperature-dependent testing method has been used, even at a slow increase in the heating rate.

#### 3.3.1 Thermal Denaturation without Solvents

Yakovchuk et al.,^58^ and Privalov^59,60^ studied the role of base-stacking and base-pairing contribution to the thermal stability DNA using calorimetry (spectrophotometer) and gel electrophoresis. Disclosing the role of hydrogen bonding between conjugate base pairs, the authors^59,60^ concluded that the formation of hydrogen bonds is an entropy-driven nonenthalpic process. The disruption of the van der Waals contacts between base pairs explained the high enthalpy values during DNA melting. The authors explained it by a disruption of apolar contacts between bases. This means that a significant part of the DNA denaturation/renaturation enthalpy is supposed to be allocated to dispersion forces.

Peter^59^ defines DNA renaturation as the sequence of the following interrelated steps: base pairing and base stacking. Both processes required proper orientation of the corresponding bases that were needed for the formation of hydrogen bonding.

#### 3.3.2 Thermal Denaturation in the Presence of Solvents

There are several directions of work devoted to the study of the thermal DNA denaturation with solvents:

Majumdar^61^ studied the melting temperature of double-stranded DNA in pure solvents and found that there is a first-order melting transition in some solvents. However, changing the quality of a solvent from good to poor led to a nonfirst-order melting curve. The author explained the denaturation process by the formation of bubbles.

Another direction of thermal DNA denaturation study was conducted by Hammouda and Worcester.^24,57^ The authors investigated the thermal denaturation transition (*T*_m_) of Salmon DNA in water and aqueous solutions of alcohols, ethylene glycol, and glycerol. The authors used UV light absorption spectroscopy with a slow heating rate and a 260 nm line to control the melting of the helix. The authors also used small-angle neutron scattering. They showed that DNA melting temperatures depend on the nature and concentrations of the solvent, and the *T*_m_ value is different from that in the case of denaturation in water. These authors explain the results of their experiments by the solvent’s ability to cross the hydrophobic sugar-rich region in DNA that behaves like a cylindrical micelle. This explanation has additional impact for understanding the process in the work of Majumdar^61^ regarding the formation of bubbles.

Bonner and Klibanov,^62^ using a similar (spectrophotometric) method, studied the influence of different synthetic and natural DNA and different solvents (DMSO, glycerol, ethylene glycol) on DNA structure and stability. Their results show a significant change in the melting temperature (*T*_m_) for all nonaqueous solvents compared to water. In addition, the authors found an increase in the *T*_m_ values for the denaturation of synthetic duplex DNA (21-mer) with an increase in the concentration of sodium chloride. In conclusion, the authors highlighted the importance of hydrophobic interactions of solvents with DNA during denaturation. This conclusion gives additional information about the mechanism of DNA denaturation in comparison to the previously discussed works.^24,57,61^

Blake and Delcourt^63^ show that the addition of formamide to the DNA solution decreases the melting temperature of the DNA by 2.4–2.96 °*C* per mole of formamide depending on the content of (G + C) in the DNA composition. This finding connects DNA structure to the denaturating ability of the surrounding solution.

The authors^63^ focused on the influence of a denaturant, while Mura^25^ studied the influence of different buffers on DNA denaturation. The study used phosphate, tris, and citrate buffers at fixed pH 7.4 at concentrations that varied systematically. They found that DNA stability increases with buffer concentration and is explicitly influenced by buffer type.

Further, Sturtevant and Geiduschek^64^ calorimetrically studied the influence of pH on the enthalpy of DNA denaturation. They concluded that the entire enthalpy change occurs in the narrow pH range associated with the macromolecular configuration change.

An important review related to the structural stability of DNA and RNA in the presence of organic solvents has been presented by S. Nakano and N. Sugimoto.^23^ The authors discussed some possible mechanisms of the influence of organic solvents on nucleic acid interactions. Among these mechanisms are the influence of the osmotic pressure and the effect of the dielectric constant on specific interactions with nucleic acid strands.

Theoretical and experimental work cited above show the influence of several factors and parameters on DNA denaturation in the presence and absence of different solvents. However, the work will continue to develop an equation connecting the rate and degree of DNA denaturation with the following interrelated parameters: the structure and state of DNA, the composition of surrounding media (solvents, buffers, and pH), and temperature. Such information helps to select the proper (co)­solvent or predict the required structure and concentration of an additive to a solution (buffer) sufficient for good DNA denaturation or double-stranded DNA stabilization at a given temperature.

In the present article, in order to compare thermal and chemical denaturation mechanisms, we will take the isolated thermal and isolated chemical denaturation processes and compare them in the absence of the other. We discuss purely chemical denaturation in the following section.

#### 3.3.3 Chemical Denaturation

Levine, Gordon, and Jenks^53^ studied the chemical denaturation of the T4 bacteriophage DNA in a constant buffer composition and temperature in the presence of 54 different denaturants. The authors mainly used the immunological method^65^ that determines only denatured DNA. This isothermal denaturation method allows the study of the pure chemical denaturation process. The authors found a critical concentration for each denaturant needed to create 50% DNA denaturation. Authors concluded that their study “provides no evidence that hydrogen bonding between the denaturing agent and DNA contributes to the denaturing effectiveness of the compounds examined.”

In order to verify this conclusion and to obtain more information on the mechanism of denaturation Baldini et al.^54^ investigated the role of alcohols on DNA denaturation. The authors studied the isothermal denaturation of calf thymus DNA as a function of the presence of alcohol in the solution. In each spectrophotometrical experiment, the authors determined the equilibrium value of the light absorbance (260 nm) to obtain the constancy (equilibrium value). Such isothermal experiments have been repeated at different temperatures. The selected method of measurement allows us to consider these data as pure chemical denaturation. The melting profiles of calf thymus DNA show that an increase in alcohol concentration and alkyl group sizes leads first to an increase and then to a decrease in the degree of DNA denaturation. Authors also measured the dependence of the compressibility of alcohol–water solution on the composition of the solution and found the curvilinear dependence of these parameters with a minimum. The authors explain these results by the mutual interconnected effect of hydrophobic and electrostatic effects. No clarification on the role of hydrogen bonds in the denaturation process was reported.

Xu, Dai, Wang, and Yang^66^ spectrophotometrically investigated DNA denaturation of high and low molecular weight molecules in the absence and presence of different concentrations of dimethyl sulfoxide (DMSO). They also analyzed changes in the configuration of DNA using atomic force microscopy (AFM technique) and dynamic light scattering (DLS). Each stage of AFM treatment of DNA samples was performed at a constant temperature, so this method of investigating the denaturation process has to be classified as chemical, not thermal.

The images on AFM-tested mica slides show DNA denaturation regions. Quantitative analysis of areas markedly denatured on the slides was performed by imaging software, and the use of the worm-like-chain model allowed the authors to reveal the dependence of DNA persistence length on the concentration of DMSO. Analysis of these data led the authors^66^ to conclude that persistent DNA length decreases with the addition of DMSO. A substantial decrease in persistent length (from 50 to 12 nm) occurs at the addition of only 3% DMSO to the solution, which is significantly below the DMSO concentration corresponding to the melting point. The addition of 1% DMSO leads to 11% denaturation of 5000 bp DNA. The authors concluded that even low DMSO concentration leads to a partial break in hydrogen bonds and weakening of the base stacking forces before the complete transition of dsDNA to ssDNA. The results also show a configuration change (increase in DNA compaction) if the DMSO concentration increases from 0.1% to 1%.

The experimental works cited above are based on a limited number of selected denaturants and do not give sufficient information on how to extend the denaturant selection or how to predict the required structure and concentration of a denaturant sufficient for complete or (if needed) for just partial DNA denaturation. The theoretical publications quoted above describe different models with assumptions that limit their predictability for denaturant selection.

All the theoretical approaches and experimental works above did not address the influence of cohesive energy density of DNA and solution, which is extremely promising in establishing the structure–performance relationship for the DNA denaturation process. We discuss these directions below.

### 3.4 Cohesion (Solubility) Parameters

The cohesion or solubility parameter represents a substance’s cohesive energy density or the energy needed to vaporize 1 mol of a substance and expand the vapor until molecules cannot interact. According to its definition, the cohesion parameter is part of the enthalpy of solubility processes and is primarily used to find/define the boundary for solute–solvent solubility. Cohesion or solubility parameter study and its practical applications in multiple areas of human activity^67,68^ have attracted the attention of many scientists in the academy and industry.^69–71^

The following are major steps in theoretical development in this area. Hildebrand^12,13^ introduced cohesion energy density parameter. Then Prausnitz, with co-workers,^72–74^ split the parameter into two components related to the forces hidden in the enthalpy of a transition process. Such splitting means introducing the additivity principle for fractional or partial cohesion parameters (summation).

Hansen^14,15,69^ using the same additivity principle approach, expanded splitting of the cohesion parameter into three fractional cohesion parameters (Hansen Solubility Parameters or HSP), based on the structure–property relationship of the solvent. These fractional parameters define the relative input or role of polar, nonpolar­(dispersion), and hydrogen cohesion forces in total cohesion energy density, which keeps molecules together in a liquid or solid state.

Karger, Snyder, and Eon^17,75^ developed a five-component set of cohesion parameters that included additional HSP physicochemical properties related to the enthalpy of a system, such as an orientation.

Authors^17,75^ used the additivity principle for the cohesive energy density parameters. The inclusion of the orientation parameter is based on the Kirkwood-Frohlich theory,^76,77^ which discussed the influence of the orientation of the electric dipoles in polar liquids. The orientation effect, according to this theory, correlates with the dielectric constant.^78^

The authors^17,75^ calculated the dispersion term from the refractive index, the orientation and induction terms from the molar volume and the dipole moment, and the product of the electron donor–electron acceptor activity in hydrogen bonds by difference.

The work on modifying or expanding fractional cohesion parameters also based on the additivity principle to the products of descriptors for the physicochemical properties of solutions and the corresponding energy-related fractional parameters specific to the solvent (“The Linear Solvation Energy Relationship”—LSER) continues.^79–86^ Review of earlier publications in this direction presented by Barton,^67^ Chapters 5 and 8.

The Panayiotou group^81–84^ thermodynamically develops and experimentally expands a method for evaluating certain fractional (partial) parameters in systems with low and significant polarity, including those with strong hydrogen-bonding interactions.

The authors confirm the additivity principle (LSER) and give the thermodynamic explanation for such additivity, specifically for hydrogen bonding. Dohnal^85^ presented the computational methodology related to a new molecular descriptor for the analysis of the solvation-related properties. Authorn^87^ suggested replacing cohesive energy densities with electrophilicity densities that incorporate the charge transfer effect as a critical contribution to the Hildebrand approach. No additional fractional cohesion parameters were proposed. We will discuss the progress related to the transition from one to five-component cohesion parameter models in more detail later in this article.

The modification of the solubility parameters continues (see, for example, refs 81 and 88 ). Authors^88^ suggested replacing cohesive energy densities with electrophilicity densities that incorporate the charge transfer effect as a critical contribution to the Hildebrand approach. No fractional cohesion parameters were proposed in this^88^ approach. We will discuss the progress related to the transition from one- to five-component cohesion parameter models in more detail below in this article, particularly the application of some of these parameters for evaluating the DNA denaturation process.

Several published theoretical articles attempted to extend the predictability of Hansen parameters beyond the solubility of polymers.^71,89,90^ We found no attempts in the literature to find the relationship between the experimentally determined cohesion parameters (HSP) and DNA denaturation in different solvent/cosolvent compositions. One of the purposes of this article is to find such a relationship.

Overall, we can conclude that extensive work has been published in the literature on DNA’s thermal and combined thermal-chemical denaturation. However, additional work is needed to establish essential factors and their relationship to pure chemical DNA denaturation. To reveal and better understand the mechanism of these processes, we use a thermodynamic approach (see below).

## 4 Theory

### 4.1 Kinetic and Thermodynamics of DNA Denaturation

The complete denaturation of DNA with high molecular mass (full transition from double-stranded, DS, helical nucleic acids to single-stranded, SS coils or DNA “melting”) is a two-stage reversible consecutive dissociation reaction. Intermediate partial denaturation (PD) is the first stage. This happens with the formation of forks and/or bubbles inside the DNA helical structure and/or coils created from the initial helix, which continue to be bound to the initial helix. This first partial denaturation stage can be expressed as

12 DS⇔PD⇔2SS

In the case of short DNA, the intermediate step can be omitted, and the process can be expressed as a simple reversible dissociation reaction

13 DS⇔2SS

The ratio of the rate constants for the forward and reverse processes *K*_a_ and *K*_b_ can be written as the equilibrium constant *K*

14 $$K=\frac{K_{a}}{K_{b}}$$

The equilibrium constant *K* for the initial step of dsDNA denaturation with the formation of partially denatured pdDNA, according to expression 12 presented earlier as eq 1 $K=\frac{\left[PD\right]}{\left[DS\right]}$ .

Here, [DS] and [PD] are the concentrations of double-stranded (helix) and denatured sections inside of each partially denatured DNA molecule. The number of DNA molecules in this stage of denaturation does not change. In this case, the equilibrium constant represents the degree of DNA denaturation.

The equilibrium constant *K*′ according to expression 12 for the final step of pdDNA denaturation with the formation of single-stranded DNA, ssDNA, is equal to eq 2: $K^{\prime }=\frac{{\left[SS\right]}^{2}}{\left[PD\right]}$ . Here [SS] is the concentration of single-stranded DNA.

The equilibrium constant *K*″ for the denaturation of dsDNA with low molecular weight to direct formation of single-stranded DNA, ssDNA, is equal to

15 $$K^{\prime \prime }=\frac{{\left[SS\right]}^{2}}{\left[DS\right]}$$

We used the thermodynamic approach to minimize the number of necessary assumptions about the mechanism of denaturation in creating our theory. Combination of the equation for Gibbs free energy change

16 ΔG=ΔH−TΔS

and the Gibbs free energy isotherm equation

17 ΔG=−RT⁡ln⁡K

leads to the Van’t Hoff or Arrhenius expression for the temperature dependence that can be applied to the degree of DNA denaturation

18 $$\ln K=A-\frac{\Delta H}{RT}$$

Here Δ*S* is entropy change, *T* is the temperature in degrees Kelvin, Δ*H* is the transition enthalpy at temperature *T*, *R* is the universal gas constant, and *A* = (Δ*S*/*R*).

The DNA denaturation process can be activated either by heating (thermal denaturation) or by adding denaturant(s), cosolvent(s), or changing buffer (pH) in the system (chemical denaturation). The mechanisms of both processes are different.

The thermal denaturation of DNA involves heating DNA, leading to breaking bonds, specifically hydrogen bonds and hydrophobic stacking attractions between the bases. Thus, thermal denaturation is an endothermic process that absorbs heat, making the net enthalpy change positive, Δ*H*_thermal_ > 0. Thermal denaturation has been proved to be endothermic by measurement of the temperature dependence of ln *K*. (See, for example, ref 53). Gibbs free energy change, in this case, is positive, Δ*G* > 0.

All processes with Δ*G* > 0, including DNA thermal denaturation, are not spontaneous.

The chemical denaturation process (at constant temperature) involves replacing the initial bonds that keep DNA in double-strand form with new “DNA + denaturant” bonds with higher energy of attraction than the previous hydrogen bonds. This leads to the separation of DNA strands and the formation of random coils or single-stranded states.

Thus, chemical denaturation is the exothermic process of releasing heat, making the total enthalpy change negative, Δ*H* < 0.

Any process has to be spontaneous in the case of Δ*G* < 0. According to eq 16, this condition takes place a) at all temperatures if Δ*S* > 0, but b) in the case of Δ*S* < 0. Spontaneous processes at Δ*H* < 0 are possible only at very low *T* when the product *T*Δ*S* is small.

DNA chemical denaturation process is spontaneous (except the case *b* above) since

19 ΔH<0;⁣ΔG<0

The chemical denaturation process in a liquid solution (with the enthalpy defined as Δ*H*_chem_) has two consecutive stages. The first stage is endothermic (with Δ*H*_endo_) when initial bonds in DNA are disrupted. The second stage is exothermic (with Δ*H*_exo_) when the open, active sites of DNA molecules interact with molecules of surrounding media, forming new bonds or restoring previous bonding inside DNA (renaturation). These two sequential stages in the denaturation process with different signs for Δ*H* have their contribution to the total enthalpy of DNA denaturation Δ*H*_chem_. The second stage cannot occur if the first stage does not occur. The total enthalpy of DNA denaturation in eq 18 in the case of two *consecutive* stages of this process can be treated as a sum of the enthalpies for both these stages

20 $$\Delta H_{chem}=\Delta H_{endo}+\Delta H_{exo}$$

and

21 $$\ln\;K=A-\frac{\Delta H_{endo}+\Delta H_{exo}}{RT}$$

Parameters Δ*H*_thermal_ and Δ*H*_endo_ are not necessarily equal. The experimental part of this paper will discuss a comparison of enthalpies of thermal and chemical denaturation processes.

Parameter *A* in eq 18 can be found at DNA melting temperature (*T*_m_) when ln *K* = 0

22 $$A={\left(\frac{\Delta S}{R}\right)}_{T=T_{m}}={\left(\frac{\Delta H}{RT}\right)}_{T=T_{m}}$$

The melting temperature *T*_m_ is the temperature for 50% DNA denaturation. Here, a molecule of DNA is partially (50%) denatured. At such conditions, eq 1 for a partially denatured state applies with [PD] = [DS] and *K* = 1.

Equation 1 describes internal DNA denaturation without splitting single-stranded molecules from the initial or partially denatured DNA unit. The denaturation process leads to an internal conformational change in DNA, decreasing the [DS] fraction in each macromolecule and increasing the [PD] fraction.

Substitution of eqs 22 to 18 with condition 19 gives an expression for the chemical denaturation process

23 $$\ln K=\frac{\Delta H}{RT}-{\left(\frac{\Delta H_{DNA}}{RT}\right)}_{T=T_{m}}$$

The eq 23 shows that the degree of DNA denaturation (K) reflects the difference in the enthalpy inside double-stranded DNA at melting temperature and the enthalpy inside the denaturant/solvent mixture at the temperature of the experiment. Parameters Δ*H* and Δ*H*_DNA_ are not equal if the temperature of the experiment is not identical to *T*_m_, but they become equal at *T* = *T*_m_.

At melting temperature, the enthalpy change in the DNA molecule is equal to the enthalpy change of the surrounding solution:(Δ*H*_DNA_)_*T*_m__ = Δ*H*_*T*_m__.eq 23 shows that the denaturing ability of a denaturant that influenced the degree of DNA denaturation (*K*), is a function of the temperature of experiment, *T*, DNA melting temperature *T*_m_, the total enthalpy (Δ*H*) of a solution at temperature of experiment and (Δ*H*)_DNA_ at melting temperature.

Combination of eqs 23 and 20 gives

24 $$\ln K=\frac{\Delta H_{endo}+\Delta H_{exo}}{RT}-{\left(\frac{\Delta H_{DNA}}{RT}\right)}_{T=T_{m}}$$

and in the case *T* = *T*_m_, when ln *K* = 0 we have

25 $$\Delta H_{chem,T_{m}}=\Delta H_{endo}+\Delta H_{exo,T_{m}}=\Delta H_{DNA,T_{m}}$$

Total enthalpy Δ*H*_chem_ for chemical denaturation will be calculated based on specific physicochemical parameters for the solutions of different chemicals used for DNA denaturation experiments at a constant temperature corresponding to DNA melting. We perform such calculations using cohesion or Hansen solubility parameters (HSP), which are discussed in the next section of this paper.

In the case of equilibrium Δ*G* = 0, and eq 16 is simplified to

26 $$T_{m}={\left(\frac{\Delta H}{\Delta S}\right)}_{T=T_{m}}$$

where *R* is the universal gas constant and *T* is absolute temperature (Kelvin). According to this equation, the melting temperature in chemical or thermal denaturation processes (and in any energy exchange processes) equals the ratio of enthalpy and entropy changes at the melting temperature. Comparison of eqs 26 and 23 shows that a single component containing the entropy term in the last of these two equations is *T*_m_.

### 4.2 Relation between Enthalpy and Cohesion Parameters for One Solvent and the Multicomponent Solutions of Denaturants

#### 4.2.1 DNA Denaturation in One-Component Liquid Systems

Hildebrand^13^ considered the change in cohesive energy per unit volume. The cohesive energy density, δ^2^ known as the Hildebrand parameter,^12^ is also called the cohesion or solubility parameter.

We applied this parameter δ^2^ to calculate the enthalpies Δ*H* of the solutions for DNA chemical denaturation. This calculation can be performed using Hildebrand equation^12^

27 $$\Delta H_{1}=\delta^{2}V+RT$$

where *V* is the molar volume of a liquid, *R* is the universal gas constant, and *T* is the absolute temperature (Kelvin).

We used this parameter known from the literature to calculate Δ*H* without heating the solution with dsDNA as a condition for chemical denaturation.

Hildebrand parameter was confined to nonassociating and nonpolar systems, but the concept has been extended to the types of systems beyond these restrictions.

Comparison of eqs 27 and 26 (which represents eq 16 at equilibrium conditions) shows the difference in the thermodynamic concepts of Gibbs and Hildebrand. They divided total molar cohesive energy (enthalpy) into different segments. Gibbs defined the enthalpy as the product of the temperature and the entropic term Δ*S*. Hildebrand defined the enthalpy as the sum of *RT* and the temperature-independent term δ^2^ *V*. We combined both these approaches, namely, combine eq 18 (which is derived from eq 16), with eq 27.

The right part of eq 27 is always positive, but the left part can be positive (the thermal DNA denaturation case) or negative (the chemical denaturation case). In the last case, eq 27, with condition 19, leads to a modified Hildebrand eq 4 Δ*H*_1_ = −δ^2^ *V* – *RT* related to the chemical type of DNA denaturation. Parameter δ^2^ *V* is known as total *cohesion or solubility parameter*.^14^

According to Hansen,^14,15^ cohesive energy is made up of a combination of additive contributions from fractional cohesion parameters (eq 7) called Hansen parameters: *δ* ^2^ = *δ*_d_^2^ + *δ*_h_^2^ + *δ*_p_^2^ where δ_d_^2^ is the dispersion or nonpolar cohesion parameter, δ_h_^2^ is the hydrogen bonding cohesion parameter, and δ_p_^2^ is the polar cohesion parameter. Methods for measuring and calculating these fractional parameters are described in^67^ and.^14^ Dimension of δ^2^ is *J*·cm^–3^; *V* is cm^3^·mol^–1^; δ^2^ *V* and *RT* are *J*·mol^–1^.

Combination of eqs 24, 25, and 20 (the case Δ*H* < 0) gives eq 5 $\ln K=\frac{{\left(\delta^{2}V\right)}_{sol}}{RT}-{\left(\frac{{\left(\delta^{2}V\right)}_{DNA}}{RT}\right)}_{T_{m}}$ . In the case of constant *T* = *T*_m_ and the same DNA in all denaturing experiments, ln *K* = 0 and this expression has the form of eq 6 ${\left(\delta^{2}V\right)}_{DNA,T_{m}}={\left(\delta^{2}V\right)}_{sol,T_{m}}$ , where subscript sol in these equations means a solution for denaturation. Parameter $\left[{\left(\delta^{2}V\right)}_{sol}\right]$ equal according to eq 4 to the term [−(Δ*H*)_chem_ – *RT*].

Using eqs 7 and 6 above, we know that at the melting temperature, the product of molar volume on the sum of each of the three fractional cohesion parameters is the same in the solution as well as in DNA.

28 $${\left[\left(\delta_{d}^{2}+\delta_{h}^{2}+\delta_{p}^{2}\right)V\right]}_{DNA,T_{m}}={\left[\left(\delta_{d}^{2}+\delta_{h}^{2}+\delta_{p}^{2}\right)V\right]}_{sol,T_{m}}$$

The total cohesion parameter of the DNA at the melting point and the total cohesion parameter of the solution are identical. We assumed that the distribution of the fractional cohesion parameters in DNA at its melting temperature are identical to that of the fractional cohesion parameters in the solution.

The modified Hildebrand parameter (with modifications shown above) applies to exothermic processes such as chemical denaturation.

#### 4.2.2 DNA Denaturation in Multicomponent Liquid Systems

A liquid media for DNA or RNA denaturation is usually a blend of aqueous solvents/denaturant(s), salts, and a buffer. We considered such systems as the media for the chemical (exothermal) type of the denaturation process. Evaluation of the effective cohesion parameters δ̅ for liquid mixture is of prime importance for selecting a proper mixture of solvents for the denaturation of nucleic acids. Hildebrand and Prausnitz^13^ assumed in developing the expression for cohesion parameter that the value of effective parameter δ̅ for a solvent mixture is volume-wise proportional to the similar parameters of its components. We assumed similarly to^13^ that for the multicomponent solution, the additivity principle for enthalpy Δ*H*_i_ and volume fraction *V*_f_i__ for each component in the solution apply. See Assumption below and compare it to eq 27.

Assumption. *We assumed that for the multicomponent solutions, we can apply the additivity principle for enthalpy* Δ*H*_i_ and *Hansen parameter* δ_i_^2^ *V*_i_ *per volume fraction* *V*_f_i__ *of each component,* eq 9: $\Delta H=\sum_{i=1}^{i=j}\Delta H_{i}V_{f_{i}}=-\left(\sum_{i=1}^{i=j}\delta_{i}^{2}V_{i}V_{f_{i}}\right)-RT$ . *Each* Δ*H*_*i*_, *V*_*i*_ *and* δ_i_^2^ *values in* eq 9 *has identical property to corresponding pure component*. The sum of all volume fractions in the system equal to one

29 $$\sum_{i=1}^{i=j}V_{f_{i}}=1$$

Combining eqs 5 and 9 above for the chemical type of denaturation in multicomponent systems, we have the following expression

30 $$\ln\;K={\left(\frac{\sum_{i=1}^{i=j}\delta_{i}^{2}V_{i}V_{f_{i}}}{RT}\right)}_{sol}-{\left(\frac{{\left(\delta^{2}VV_{f}\right)}_{DNA}}{RT}\right)}_{T_{m}}$$

where ${\left(V\times V_{f}\right)}_{DNA,T_{m}}$ is the product of molar volume and volume fraction of DNA at melting temperature.

Based on eq 30, we have developed a method to estimate the type and required concentration of different denaturants to create a solution with perfect denaturing power. Such denaturant(s) should have a high value of the fractional Hansen solubility parameter related to the limiting enthalpic component needed for denaturation and have sufficient solubility in a targeted solvent/cosolvent mixture at the required temperature. For example, the denaturant selected for effective DNA chemical denaturation should have a high value of the partial cohesion parameter δ_h_ related to hydrogen bonds and high solubility to create a large value *V*_f_.

In the case of constant *T* = *T*_m_ and the same type and concentration of DNA in all denaturing experiments ln *K* = 0 and eq 30 has a form

31 $${\left(\delta^{2}VV_{f}\right)}_{DNA,T_{m}}={\left(\sum_{i=1}^{i=j}\delta_{i}^{2}V_{i}V_{f_{i}}\right)}_{sol,T_{m}}$$

The expression [Disp-formula eq31] shows that there is a corresponding interaction between DNA and other components in the solution. This allows us, for the case of ln *K* = 0, when the equilibrium between DNA and solution is established (eq 31), to determine the thermodynamic parameters of DNA by measuring these parameters of the surrounding solution.

We have developed a theory describing the chemical denaturation of DNA as a reversible first-order reaction. The theory links the degree of DNA denaturation with Hildebrand, Hansen, and KSE cohesion parameters, concentration (volume fraction) of a denaturant, and temperature.

## 5 Analysis of Experimental Data

We analyze the experimental data published by other researchers using our theoretical framework to support the theory. This is necessary for independent verification of the theory.

### 5.1 Materials and Methods

#### 5.1.1 DNA and Its Denaturation

The primary data set of 31 chemicals for DNA denaturation was tested by.^53^ The following DNA types have been tested:^53^ T4 Bacteriophage, Diplococcus pneumoniae, *Bacillus subtilis*, calf thymus, S. coli B, *Serratia marcescens*, *Pseudomonas aeruginosa*, and Streptomyces *viridochromogenes*. The majority of the experiments were done on the T4 Bacteriophage DNA at a concentration of 16μg·mL^–1^ in a buffer containing 0.005 M Tris, and 10^–3^ M EDTA, pH 7.6, and incubated for 30 min at 73 °*C*. Then, the DNA samples were cooled rapidly on ice. Following DNA denaturation, an immunologic method was used to identify solely denatured DNA.^53^ The logarithmic plot of the ratio of denatured to native DNA against the concentration of denaturing agents was used to calculate the concentrations of denaturing agents needed to give 50% denaturation of DNA under these conditions. The thermal DNA denaturation has also been studied using immunological and optical (refractometry, relative absorbance of 260 nm) methods. Experiments were performed^53^ to study DNA denaturation as a function of DNA concentration ranging from 0.9 μg·mL^–1^ to 30 μg·mL^–1^. The present article provides new insight into experimental data cited above^53^ by combining with effective cohesion parameters of solutions using a newly developed method below.

#### 5.1.2 Method of Calculation of Physicochemical Parameters for DNA Chemical Denaturation

The method of calculation of the total and fractional enthalpies for DNA three-set cohesion parameters is presented in the form of eqs 34–37 and for five-set cohesion parameters as^38–42^ . The experimentally determined and published values of cohesion parameters for all tested denaturants and water were adapted from the references in Tables 1 and 3.

**Table 1:** Denaturant Concentration (*M*) Giving 50% Denaturation of T4 Bacteriophage DNA in Buffer Solution at 73 °C and Hansen Parameters of Denaturants at 25 °C^a^

#	denaturants	*M* (*) mol·L^–1^	*V* cm^3^·mol^–1^	references for cohesion data
	**Alcohols**			
1	methyl alcohol	3.5	40.7	(A), pg 101.
2	ethyl alcohol	1.2	58.5	(A), pg 101.
3	isopropyl alcohol	0.9	76.8	(A), pg 101.
4	*n*-propyl alcohol	0.54	75.2	(A), pg 101.
5	allyl alcohol	0.5	68.4	(A), pg 101.
6	*sec*-butyl alcohol	0.62	92.4	(A), pg 123
7	isobutyl alcohol	0.45	92.8	(A), pg 101.
8	*n*-butyl alcohol	0.33	91.9	(A), pg 101.
9	*n*-butyl alcohol	0.33	91.5	(A), pg 123
10	*tert*-amyl alcohol	0.39	109.6	(A), pg 101.
11	ethylene glycol	2.2	56	(***)
12	glycerol	1.8	73.2	(A), pg130
13	glycerol	1.8	73.2	(***)
14	cyclohexyl alcohol	0.22	106	(A), pg 101.
15	benzyl alcohol	0.09	103.7	(A), pg 123
16	phenol	0.08	87.5	(A), pg 102
	**Other Cyclic Compounds**			
17	aniline	0.08	92	(A), pg 123
18	pyridine	0.09	80.9	(A), pg 135
19	1,4-dioxane	0.64	85.7	(A), pg 98
20	butyrolactone	0.55	76.7	(A), pg 124
	**Amides**			
21	formamide	1.9	39.8	(A), pg 100
22	*N*-ethylformamide	1	76.8	(A), pg 253
23	*N*,*N*-dimethylformamide	0.6	77	(A), pg 100
24	acetamide	1.1	60.8	(B)
25	acetamide	1.1	51	(A), pg 268
26	*N*-ethylacetamide	0.88	100.5	(A), pg 253
27	*N*,*N*-dimethylacetamide	0.6	93	(A), pg 142
28	propionamide	0.62	69.9	(***)
29	propionamide	0.62	78.9	(B)
30	butyramide	0.46	95.4	(***)
31	thioacetamide	0.62	68.3	(***)
	**Ureas & Others**			
32	urea	1	44.9	(A), pg 108
33	thiourea	0.41	49.8	(***)
34	acetonitrile	1.2	52.6	(***)
35	acetonitrile	1.2	53	(A), pg 123
36	water	NA	18	(A), pg (**)

a The References column shows which 3-set cohesion parameters were used in our calculations. (A) Barton Handbook;^67^ (B) Hansen Handbook;^69^ (*) Adapted from the ACS publication ref 53; (**) Average value of Hansen parameters for water calculated from ref 67 pgs.103, 107, 115, 118, 137, 142, 288. (***) https://www.stevenabbott.co.uk/practical-solubility/hsp-basics.php.

**Table 2:** Averages for Total and Fractional Entropies and Enthalpies for Solutions of Chemicals at the Concentration That is Required to Give 50% Denaturation of T4 Bacteriophage DNA at 346 K for Three-Component Cohesion Parameters

properties for	(Δ*H*) and (Δ*S*) for	(Δ*H*) and (Δ*S*) for	(Δ*H*) and (Δ*S*) for	(Δ*H*) and (Δ*S*) for total
solubility parameters	δ_d_	δ_h_	δ_p_	δ
(Δ*H*), J·mol^–1^ ^a^	6670.5	28106.2	11350.0	42038.0
% of total enthalpy	15.9%	66.9%	27.0%	100.0%
(Δ*S*), J·mol^–1^·K^–1^ ^b^	19.28	81.23	32.80	121.50

a The enthalpy values are the averages from the data presented in Figure 1.

b Entropy calculated from eq 26.

**Table 3:** Denaturant Concentration (M) Giving 50% Denaturation of T4 Bacteriophage DNA in TRIS–EDTA Buffer Solution pH = 7.6 at 73 °C and Five-Component Cohesion Parameters for Denaturants at 25 °C^a^

#	denaturants	*M* (*) mol·L^–1^	*V* cm^3^·mol^–1^	References for cohesion data
	**Alcohols**			
1	methyl alcohol	3.5	40.7	(A), pg 76
2	ethyl alcohol	1.2	58.5	(A), pg 76.
3	*n*-propyl alcohol	0.54	75.2	(A), pg 77.
4	*n*-propyl alcohol	0.54	75.2	(A), pg 76.
5	*sec*-butyl alcohol	0.62	92.4	(A), pg 77.
6	isobutyl alcohol	0.45	92.8	(A), pg 77.
7	*n*-butyl alcohol	0.33	91.9	(A), pg 77.
8	ethylene glycol	2.2	56	(A), pg 76
9	cyclohexyl alcohol	0.22	106	(A), pg 77.
10	phenol	0.08	92	(A), pg 76.
	**Other Cyclic Compounds**			
11	pyridine	0.09	80.9	(A), pg 76.
12	1,4-dioxane	0.64	85.7	(A), pg 76.
13	gamma-butyrolactone	0.55	76.7	(A), pg 76.
	**Amides**			
14	*N*,*N*-dimethylformamide	0.6	77	(A), pg 76.
15	*N*,*N*-dimethylacetamide	0.6	92	(A), pg 76.
	**Others**			
16	acetonitrile	1.2	53	(A), pg 76.
17	water		18	(A), Pgs (**)

a The References column shows which 5-set cohesion parameters were used in our calculations. (*)­Values of M adapted from the ACS publication ref 53 Lawrence Levine, Julius A. Gordon, and W. P. Jencks. (**)­Average cohesion parameters for water calculated from ref 67, pgs. 76, 77. (A) Barton Handbook.^67^

### 5.2 Results and Discussion

#### 5.2.1 DNA Denaturation Analysis Using Hansen Cohesion Parameters to Support the Theory

To support the theory above, we analyzed published experimental data containing information needed for inclusion in eqs 31 and 30 and calculation of Δ*H*_sol_.

Values of experimentally determined Hildebrand parameters δ and molar volumes *V* (at 25^◦^ *C*) for denaturants shown in Table 1 have been adapted from literature refs 67 and 69 .^91,92^ These data have been used to calculate enthalpies for each solution composition.

Note that the values of δ and *V* separately depend on temperature, but the function δ^2^ *V* (Hansen solubility parameter, HSP) according to eq 27 is temperature independent. Thus, we can apply HSP calculated for 25 °C to the actual experimental temperature for DNA denaturation.

Levine, Gordon, and Jencks’s^53^ studied at constant temperature (73 °C) the effectiveness of a broad spectrum of chemicals as denaturing agents (see Table 1). These authors show the dependence of the denaturation degree, *K*, for *T*_4_ bacteriophage DNA from the concentration of denaturants *M*, expressed in mol·L^–1^. They found the concentration for each additive *M*_T_m__ corresponding to 50% DNA denaturation that equals *K* = 1. These data are also presented in Table 1.

The constancy of the total and fractional enthalpies in Figure 1 for systems in Table 1 indicates that there is no phase separation in these water and denaturant mixtures at the given temperature and concentrations of denaturants.

**Figure 1:**
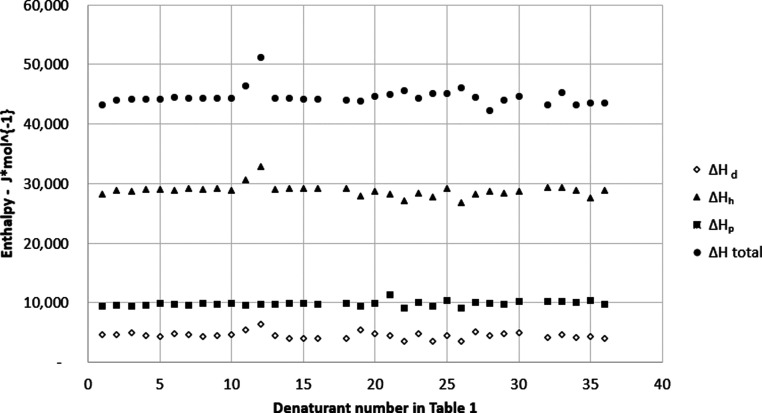
Total and fractional enthalpies for solutions of chemicals at the concentration that is required to give 50% denaturation of T4 bacteriophage DNA at 346 K for three-component cohesion parameters for denaturants of different structures described in Table 1.

We defined ${\left(\delta^{2}VV_{f}\right)}_{sol,T_{m}}$ as parameter *L*_i_. Verification of eq 30 and 31 can also be done by measurements of the term from the right part of eq 31

32 $${\left(\sum_{i=1}^{i=j}\delta_{i}^{2}V_{i}V_{f_{i}}\right)}_{sol,Tm}={\left(\sum_{i=1}^{i=j}L_{i}\right)}_{sol,T_{m}}$$

keeping the left part of eq 30 constant.

Temperature constancy is an important condition for such experiments where the same type and DNA concentration are used for denaturation in different solvents. At the melting point, the parameter *L*_*i*_ will remain the same for both DNA and the solution.

The authors^53^ measured the degree of denaturation of T4 bacteriophage with a constant concentration in solutions of different denaturants at constant temperature *T* = 73 °*C*. Each of these solutions has four components: a denaturant (1), 0.05 M Tris buffer (2), 0.001 M EDTA (3), and water (w). The expression 32 can be written accordingly as *L*_1_ + *L*_2_ + *L*_3_ + *L*_w_. Because (*L*_1_ + *L*_w_) ≫ (*L*_2_ + *L*_3_) expression [Disp-formula eq32] is simplified as

33 $${\left(\delta_{1}^{2}V_{1}V_{f_{1}}+\delta_{w}^{2}V_{w}V_{f_{w}}\right)}_{sol,T_{m}}={\left(L_{1}+L_{w}\right)}_{sol,T_{m}}=L$$

and *L*_2_ and *L*_3_ are negligible because of their very low volume fraction, *V*_f_.

eqs 4 and 7 together with [Disp-formula eq33] give expressions for enthalpy Δ*H* using full and fractional Hansen parameters describing chemical denaturation of DNA at its melting temperature when ln *K* = 0

34 $${\left(-\Delta H=\delta_{1}^{2}V_{1}V_{f_{1}}+\delta_{w}^{2}V_{w}V_{f_{w}}+RT\right)}_{T_{m}}$$

35 $${\left(-\Delta H_{h}=\delta_{h_{1}}^{2}V_{1}V_{f_{1}}+\delta_{h_{w}}^{2}V_{w}V_{f_{w}}+RTF_{h}\right)}_{T_{m}}$$

36 $${\left(-\Delta H_{d}=\delta_{d_{1}}^{2}V_{1}V_{f_{1}}+\delta_{d_{w}}^{2}V_{w}V_{f_{w}}+RTF_{d}\right)}_{T_{m}}$$

37 $${\left(-\Delta H_{p}=\delta_{p_{1}}^{2}V_{1}V_{f_{1}}+\delta_{p_{w}}^{2}V_{w}V_{f_{w}}+RTF_{p}\right)}_{T_{m}}$$

where Δ*H*_d_ is the fraction of enthalpy related to dispersion or nonpolar forces, Δ*H*_h_ is the fraction of enthalpy related to hydrogen bonding forces, and Δ*H*_p_ is the fraction of enthalpy related to polar forces. $F_{h}=\frac{L_{h}}{\left(L_{d}+L_{h}+L_{p}\right)}$ , $F_{d}=\frac{L_{d}}{\left(L_{d}+L_{h}+L_{p}\right)}$ , and $F_{p}=\frac{L_{p}}{\left(L_{d}+L_{h}+L_{p}\right)}$ . Note that *F*_d_ + *F*_h_ + *F*_p_ = 1.

All data presented in Figure 1 corresponds to a constant temperature of experiments *T* = *T*_m_ and according to eq 26 must have constant Δ*H*_Tm_ values, which is shown in Figure 1.

The selection of the wide range of different denaturants with significant differences in physicochemical parameters such as δ (20.5 to 47.85), *V* (18 to 110 cm^3^·mol^–1^), and *V*_f_ (0.01 to 0.14) do not change the constancy of Δ*H* values at *T* = *T*_m_ demonstrated in Figure 1. This is the second proof of the correctness of eqs 30 and 31 that validates the theory. We can conclude that the Hildebrand equation applies to the chemical denaturation process.

The average values for the total and fractional enthalpies for a solution at *T*_m_ equal the enthalpies of the T4 bacteriophage DNA. These averages are presented in Table 2. Based on this data, we calculated the fractions of enthalpy for dispersion, polar, or hydrogen types of DNA bonding.

Results show that for chemical denaturation of T4 bacteriophage DNA, the effect of hydrogen bonding is the dominant part of enthalpy (67%). Table 2 also indicates that dispersion forces have the lowest influence on enthalpy (11%). This direct experimental data is opposite to the conclusions presented by Dragan, Crane-Robinson, and Privalov (see introduction^42,59^ ) related to the thermostability of DNA in the absence of denaturants.

Their results^42,59^ show that dispersion (apolar, van der Waals) forces are the main portion of the enthalpy. These forces provide around 60% of Gibbs free energy for DNA denaturation. Estimation^69^ of fractional DNA cohesion parameters for polyelectrolyte (DNA) as averages for four DNA bases gives δ_d_ = 19.8, δ_h_ = 12.3, δ_p_ = 12.2. Corresponding fractional δ^2^ values are 392.04, 151.29, 148.84. These δ^2^ values are proportional to the enthalpy of denaturation. Thus the fractional enthalpy of DNA denaturation equals Δ*H*_d_ = 57%, Δ*H*_h_ = 22%, and Δ*H*_p_ = 22% i.e. the main portion (57%) of the enthalpy of the DNA thermal denaturation process belongs to dispersion forces that correspond to the conclusion of authors.^42,59^ However, calculating the cohesive parameters of the polyelectrolyte as an average from four DNA bases is unclear because it did not consider the role of phosphate and other polar groups in a polyelectrolyte.

The opposite observations discussed above about the role of dispersion and hydrogen forces in the DNA denaturation process prove the differences in chemical and thermal denaturation mechanisms.

Analysis of eq 26 showed that the only component containing the entropic term in the equation for chemical denaturation is *T*_m_. The parameter *T*_m_ is included in eqs 34, 36, 35, 37. This leads to the conclusion that the chemical DNA denaturation process has both an enthalpic and entropic nature (See eq 26.)

Different DNA structures bond differently, and their resistance to the denaturation process can be different than in the case of T4 bacteriophage DNA. Levine^53^ studied the chemical denaturation of six different types of DNA (including T4 bacteriophage) and showed that the stabilities of these different DNA molecules against denaturation in the presence of urea or pyridine increases with increasing guanine (G) plus cytosine (C) content of the DNA. This phenomenon can be explained by comparison of corresponding enthalpy changes. Mo^93^ shows that the GC pair has a binding energy (−25.4 kcal·mol^–1^) that is twice the binding energy of the adenine (A) and thymine (T) pair (−12.4 kcal·mol^–1^). This is why these different DNA molecules respond differently toward chemical denaturation in the presence of urea or pyridine and why a higher denaturant concentration is needed to melt DNA with higher GC content. According to eqs 34, 35, 36, 37, an increase in *V*_f_ of denaturants leads to an increase in the denaturing power of the solution needed to achieve 50% of DNA denaturation.

Levine et al.^53^ also studied the effect of the concentration of T4 bacteriophage DNA on the degree of its denaturation. The authors^53^ show that a higher degree of DNA denaturation in 1.0 M urea at 346 K occurred in more dilute solutions for DNA. This observation fits the prediction based on analysis of eq 30. An increase in DNA concentration, *V*_f_, in the second term on the right-hand side of eq 30, leads to a decrease in the ln *K* term. Thus, observing the effect of DNA concentration on denaturation further confirms our theory.

#### 5.2.2 DNA Denaturation Analysis Using Expanded Cohesion Parameters

Applying fractional cohesion parameters to calculate the enthalpies of chemical denaturation revealed the forces affecting this process. We applied Hansen’s concept of three-component fractional cohesive parameters to consider the influences of the dispersion or nonpolar interaction, the hydrogen bonding, and the polar interaction in the analysis of the chemical denaturation of DNA.

Karger, Keller, Snyder, and Eon developed^16–19^ a set of five-component fractional cohesive/solubility parameters: δ_d_ = dispersion, δ_o_ = dipole orientation, δ_i_ = dipole induction, δ_a_ = hydrogen bonding related to proton-donor or acid (a), δ_b_ = hydrogen bonding related to proton-acceptor or base (b), and δ = total cohesive or solubility parameter. Parameter δ_o_ is related to the orientation effect between two molecules with a permanent dipole moment. Parameters δ_a_ and δ_b_ are presented by authors^16–19,75^ to define nonsymmetrical electron-donor and electron-acceptor properties of two components (DNA and denaturant) with different roles. This issue has been explained in terms of Lewis acid–base cohesion parameters.^94^

The relationship among these parameters was presented in eq 8. This equation shows fractional cohesion parameters representing the acting forces affecting a solubility process. The methods of the experimental determination for each of these parameters are described in ref 67 page 75. The expressions for the enthalpies of the DNA denaturation process for five-component cohesion parameters shown below have been derived similarly to eqs 34, 36, 35, 37 for three-component cohesion parameters. The eq 34 for total enthalpy value is identical for three and five-component cases.

38 $${\left(-\Delta H_{d}=\delta_{d_{1}}^{2}V_{1}V_{f_{1}}+\delta_{d_{w}}^{2}V_{w}V_{f_{w}}+RTF_{d}^{\prime }\right)}_{T_{m}}$$

39 $${\left(-\Delta H_{o}=\delta_{o1}^{2}V_{1}V_{f_{1}}+\delta_{ow}^{2}V_{w}V_{f_{w}}+RTF_{o}^{\prime }\right)}_{T_{m}}$$

40 $${\left(-\Delta H_{i}=\delta_{i_{1}}^{2}V_{1}V_{f_{1}}+\delta_{iw}^{2}V_{w}V_{f_{w}}+RTF_{i}^{\prime }\right)}_{T_{m}}$$

41 $${\left(-\Delta H_{a}=\delta_{a_{1}}^{2}V_{1}V_{f_{1}}+\delta_{aw}^{2}V_{w}V_{f_{w}}+RTF_{a}^{\prime }\right)}_{T_{m}}$$

42 $${\left(-\Delta H_{b}=\delta_{b_{1}}^{2}V_{1}V_{f_{1}}+\delta_{bw}^{2}V_{w}V_{f_{w}}+RTF_{b}^{\prime }\right)}_{T_{m}}$$

where $F_{d}^{\prime }=\frac{L_{d}^{\prime }}{\left(L_{d}^{\prime }+L_{o}^{\prime }+L_{i}^{\prime }+L_{a}^{\prime }+L_{b}^{\prime }\right)}$ , $F_{o}^{\prime }=\frac{L_{o}^{\prime }}{\left(L_{d}^{\prime }+L_{o}^{\prime }+L_{i}^{\prime }+L_{a}^{\prime }+L_{b}^{\prime }\right)}$ , $F_{i}^{\prime }=\frac{L_{i}^{\prime }}{\left(L_{d}^{\prime }+L_{o}^{\prime }+L_{i}^{\prime }+L_{a}^{\prime }+L_{b}^{\prime }\right)}$ , $F_{a}^{\prime }=\frac{L_{a}^{\prime }}{\left(L_{a}^{\prime }+L_{o}^{\prime }+L_{i}^{\prime }+L_{a}^{\prime }+L_{b}^{\prime }\right)}$ , and $F_{b}^{\prime }=\frac{L_{b}^{\prime }}{\left(L_{d}^{\prime }+L_{o}^{\prime }+L_{i}^{\prime }+L_{a}^{\prime }+L_{b}^{\prime }\right)}$ . Note that *F*_d_^′^ + *F*_o_^′^ + *F*_i_^′^ + *F*_a_^′^ + *F*_b_^′^ = 1.

Expressions above for total and fractional enthalpies Δ*H* describe the chemical denaturation of DNA at its melting temperature when ln *K* = 0.

The constancy of the total and fractional enthalpies in Figure 2 for systems in Table 3 indicates that there is no phase separation in these water and denaturant mixtures at the given temperature and concentrations of denaturants. This result is similar to that of three fraction cohesion parameter systems (see Figure 1).

**Figure 2:**
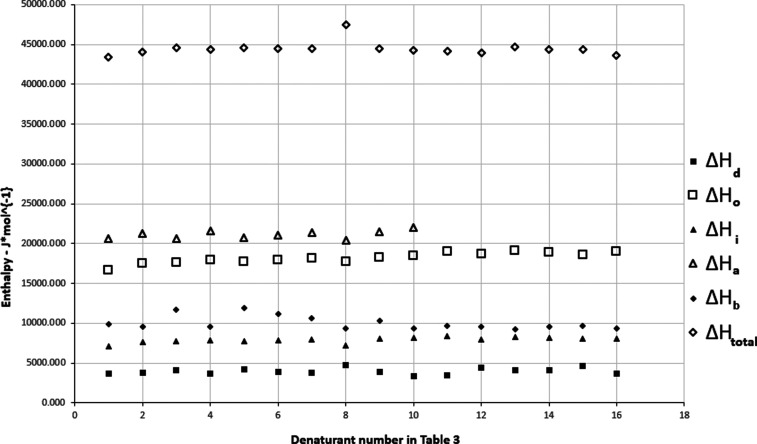
Total and fractional enthalpies for solutions of chemicals at the concentration that is required to give 50% denaturation of T4 bacteriophage DNA at 346 K for five-component cohesion parameters for denaturants with different structures shown in Table 3.

Even though there are significant differences in physicochemical parameters such as δ (20.7 to 47.85), V (18 to 106 cm^3^·mol^–1^), and *V*_f_ (0.01 to 0.14), all total and fractional Δ*H*_*T*m_ values stay constant. This again proves the correctness of eq 31 that validates the theory for chemical denaturation.

The average values from Figure 2 of the total and each of the five fractional enthalpies for the denaturation process Δ*H* at *T* = *T*_m_ are presented in Table 4. Based on this data, we calculated the fractions of the enthalpy responsible for specific mechanisms of DNA denaturation. Results show that for the chemical type of T4 bacteriophage DNA denaturation, melting is strongly related to the orientation effect (30%) as well as the disruption of hydrogen bonds in DNA according mainly to the proton-donor role of DNA (35%). Proton-acceptor role Δ*H*_b_ is significantly lower (16%).

**Table 4:** Total and Fractional Entropies and Enthalpies for the Concentration of Denaturant Required to Give 50% Denaturation of T4 Bacteriophage DNA at 346 K for Five-Component Cohesion Parameters

properties for solubility parameters	(Δ*H*) and (Δ*S*) for total	(Δ*H*) and (Δ*S*) for	(Δ*H*) and (Δ*S*) for	(Δ*H*) and (Δ*S*) for	(Δ*H*) and (Δ*S*) for	(Δ*H*) and (Δ*S*) for
	δ	δ_d_	δ_°_	δ_i_	Δ_a_	δ_b_
(Δ*H*), J·mol^–1^ ^a^	44431.53	3867.78	18051.30	7799.44	21128.89	9885.53
% of total enthalpy		6%	30%	13%	35%	16%
(Δ*S*), J·mol^–1^·K^–1^ ^b^	128.41	11.18	52.17	22.54	61.07	28.57

a The enthalpy values are the averages from the data presented in Figure 2.

b Entropy at equilibrium in the system calculated as Δ*S* = Δ*H*/*T*_m_.

Comparison of eqs 7 and 8 for three-component and five-component cohesion parameters leads to the conclusion that

43 $$\delta_{h}^{2}=2\delta_{a}\delta_{b}$$

This equation and the data in Table 4 confirm our earlier conclusion from the analysis of Table 3 that the dominant role in the enthalpy of DNA denaturation is the disruption of hydrogen bonds. Five-component analysis reveals details of this mechanism related to nonsymmetrical electron-donor and electron-acceptor properties of two components (DNA and denaturant) with different roles.

Analysis of Table 4 shows the significant role of the orientation in the DNA denaturation process related to enthalpy. Sinanoglu and Abdulnur^50^ show that DNA dipoles orienting the solvent dipoles are related to the entropic contribution to the solvent’s free energy (Δ*G*). Thus, our data above and the data of authors^50^ show that both the enthalpy and the entropy contribute to the orientation effect as part of the DNA denaturation process. This effect usually takes place as a result of two permanent dipole–dipole interactions. The connection of the orientation and induction cohesive components to such electrostatic parameters as dipole moment, polarizability, and ionization potential has been discussed by Gardon,^95,96^ Keller, Karger, and Snyder^19^ and Munafo, Buchman, Ho, and Kesselring.^97^ Parameter δ_o_ of the five-component cohesion parameter set is related to δ_p_ from the three-component cohesion parameter set. Comparing eqs 7 and 8 leads to the conclusion that

44 $$\delta_{p}^{2}=\delta_{o}^{2}+2\delta_{i}\delta_{d}$$

The connection of the orientation and induction effects to the polar cohesion parameter has been discussed in the articles of.^19,95–97^ The presence of the dispersion forces term in the polar cohesion parameter has historical roots. The polar-nonpolar solvent interaction had been studied mainly by Prauznitz and co-workers^13,72–74^ during the development of an expression for the two-component cohesion parameter (before Hansen developed his three-component system) where ${\left(\delta_{o}^{2}\right)}^{\prime }$ is only used in Prauznitz’s equation and is not equal to δ_o_^2^ in eq 44.

Weimer and Prausnitz^73^ suggested incorporating the product of polar and nonpolar components δ_d_ and ${\left(\delta_{o}\right)}^{\prime }$ into the theory. They noted a possible induction mechanism of polar-nonpolar interaction during the collision between one carbon–carbon bond with a polar molecule. This interaction is possible due to the known induction/polarizability of the carbon–carbon bond being attacked by a polar molecule. Another noninduction mechanism/role of orientation effect on the DNA renaturation process has been discussed by Privalov regarding base pairing and base stacking in the absence of solvents.^59^ Both these processes required proper orientation of corresponding bases that were needed for the formation of hydrogen bonding. The incorporation of solvents should influence this orientational effect by a thermodynamical factor (as competitors for active sites on the bases).

The five-component cohesion parameter theory,^19,97^ and eq 8 expand previous knowledge related to the nature of partial cohesion parameters and allow us to obtain a deeper understanding of the physicochemical processes in DNA denaturation.

Overall, we can conclude that experimental data for DNA chemical denaturation has confirmed the theory developed in the present article and is based on the combination of Gibbs and Hildebrand’s expanded thermodynamic approaches. This theory allows the calculation of both the enthalpy and the entropy at 50% DNA denaturation. The expression for the entropy of this process at DNA melting temperature can be obtained by the combination of eqs 26 and 9

45 $${\left(\Delta S\right)}_{T_{m}}=\frac{\sum_{i=1}^{i=j}\delta_{i}^{2}V_{i}V_{f_{i}}}{T_{m}}+R$$

#### 5.2.3 Change of Fractional Cohesion Parameters with Change of Denaturant Chemical Structure

The influence of chemical structure on the change in cohesion parameter can be shown by the comparison of fractional cohesion parameters for different denaturant groups and within specific groups:

1. Aliphatic alcohols from methanol to butanol (Table 1, denaturants 1–9)
2. Denaturants with one carbon and increasing amounts of hydroxyl groups: methanol, ethylene glycol, and glycerol (Table 1, denaturants 1, 11, and 12/13)
3. Urea (Table 1, denaturant 32)

For normal aliphatic alcohols (Table 1, denaturants 1, 2, 4, 8, and 9), there is a decrease of hydrogen bonding and polar forces as the carbon chain increases in length, and dispersion forces remain practically constant.

Comparing cohesion parameters of all aliphatic alcohols to the corresponding fractional parameters of urea (denaturant 32) shows that urea has higher values for these parameters related to dispersion forces, hydrogen bonding, and polar forces. The total cohesion parameter for urea is also higher than any of the total cohesion parameters of alcohols. It is known that urea is a much stronger denaturant than alcohols, so there is a correlation between the denaturing strength of chemicals and the cohesive properties of those chemicals. In addition, the higher denaturing strength of urea is due to its ability to act as both a substantial hydrogen bond donor and acceptor. The denaturation process involves the replacement of the water-DNA bonding with the denaturant-DNA bonds. Thus, the cohesive energy between denaturant and DNA should be comparable to that of water with DNA. The data in Table 1 (denaturant 36) shows that water has high values for all fractional and total cohesion parameters. Partial replacement of the hydration shell on DNA with urea is facilitated by an increase in its concentration up to 8 mol·L^–1^.

In addition, urea can directly form multiple hydrogen bonds with the polar groups on nucleic acid bases. The alcohols are also capable of hydrogen bonding, but form weaker interactions than urea (lower cohesive energy density, Table 1), so their ability to replace water from its interaction with DNA is much lower than for urea.

Increasing the number of hydroxyl groups for alcohols (Table 1, denaturants 1, 11, and 12/13) leads to an increase in the value of the hydrogen bonding-related cohesion parameter. This leads to an increased denaturing strength of the denaturant, as indicated by the concentration of the denaturant at which it denatures the DNA to 50% denaturation; as the cohesion parameter for hydrogen bonding increases, the concentration required to reach 50% denaturation decreases.

All these comparisons demonstrate that a shift in the hydrophilic/hydrophobic balance in a denaturant results in changes in the cohesive properties of the denaturant and its denaturing power.

#### 5.2.4 Electrostatic Repulsion Forces Participating in Maintaining dsDNA Helix

The presence of highly charged phosphate groups is the reason for electrostatic repulsion between two strands in dsDNA, compensated by the attraction forces described above. All known fractional cohesion parameters are related to different attraction forces, and none define the contribution of repulsive (electrostatic) forces. So, until now, it has been unknown how to directly measure or even estimate the part of electrostatic repulsive forces responsible for maintaining the dsDNA helix. However, the stability of the dsDNA helix at equilibrium results from a balance between electrostatic repulsive forces and the sum of attractive forces defined by measurable or known fractional cohesion parameters described and evaluated earlier in the present article. Based on this balance, we can conclude that in the case of chemical denaturation, the total cohesion parameter for attraction forces (positive value) is equal to and quantitatively describes electrostatic repulsion forces inside the dsDNA helix (the negative value). This conclusion is valid for 50% DNA denaturation when, according to expressions 7, 28 and 31, cohesion parameters in solution correspond to similar parameters in DNA.

#### 5.2.5 Method for Revealing and Evaluating of Attraction and Repulsion Forces in DNA

The method uses the following steps:

- Select the targeted DNA or another system with controllable denaturation (object).
- Select several solutions with chemicals suitable for denaturing the object at the temperature needed for application. The cohesion parameters for selected chemicals should be known.
- Measure the concentration of a denaturant needed for 50% of the object’s denaturation at a constant temperature.
- Calculate total and fractional enthalpies for the object according to eqs 32 and 34–42, or similarly modified equations (if there are more significant components in the solution).
- Use the results to reveal the influence of repulsive and different attractive forces at 50% denaturation of the object.
- Test the denaturation of different objects (DNA or other systems with controllable denaturation) in solutions with varying compositions (denaturants, buffer, pH, etc.) to select the object and/or optimize the composition of the solution suitable for your needs.

Sections 3.2 list several possible objects for this method’s application. It should also be of interest to Supramolecular chemistry,^98^ and related products.^99,100^

The total cohesion parameter of the DNA at its melting point and the sum of the total cohesion parameters for the components of the solution are identical. We assumed that the distribution of the fractional cohesion parameters inside DNA at its melting temperature are identical to that of the fractional cohesion parameters for the components in the solution.

#### 5.2.6 Comparison of Thermal and Chemical Types of DNA Denaturation

As shown above, the mechanisms of chemical and thermal denaturation processes are different. Thermal denaturation is an endothermic process, and chemical denaturation is an exothermic process. This endothermic type of denaturation can be demonstrated using recalculated experimental data of^53^ as the temperature dependence of ln *K* with a negative slope of the Van’t Hoff plot. The data is presented in Figure 3 and 4.

**Figure 3:**
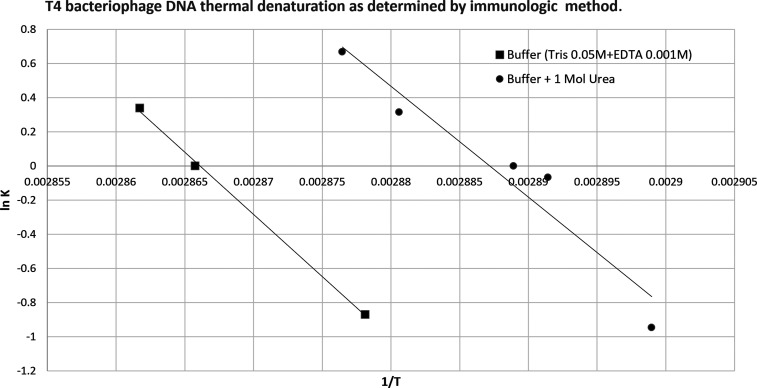
Thermal stability of DNA as determined by immunologic method. Denaturation has been performed in the presence and absence of Urea in Tris/EDTA buffer. Recalculated from the ACS publication ref 53 where *T* is in Kelvin.

**Figure 4:**
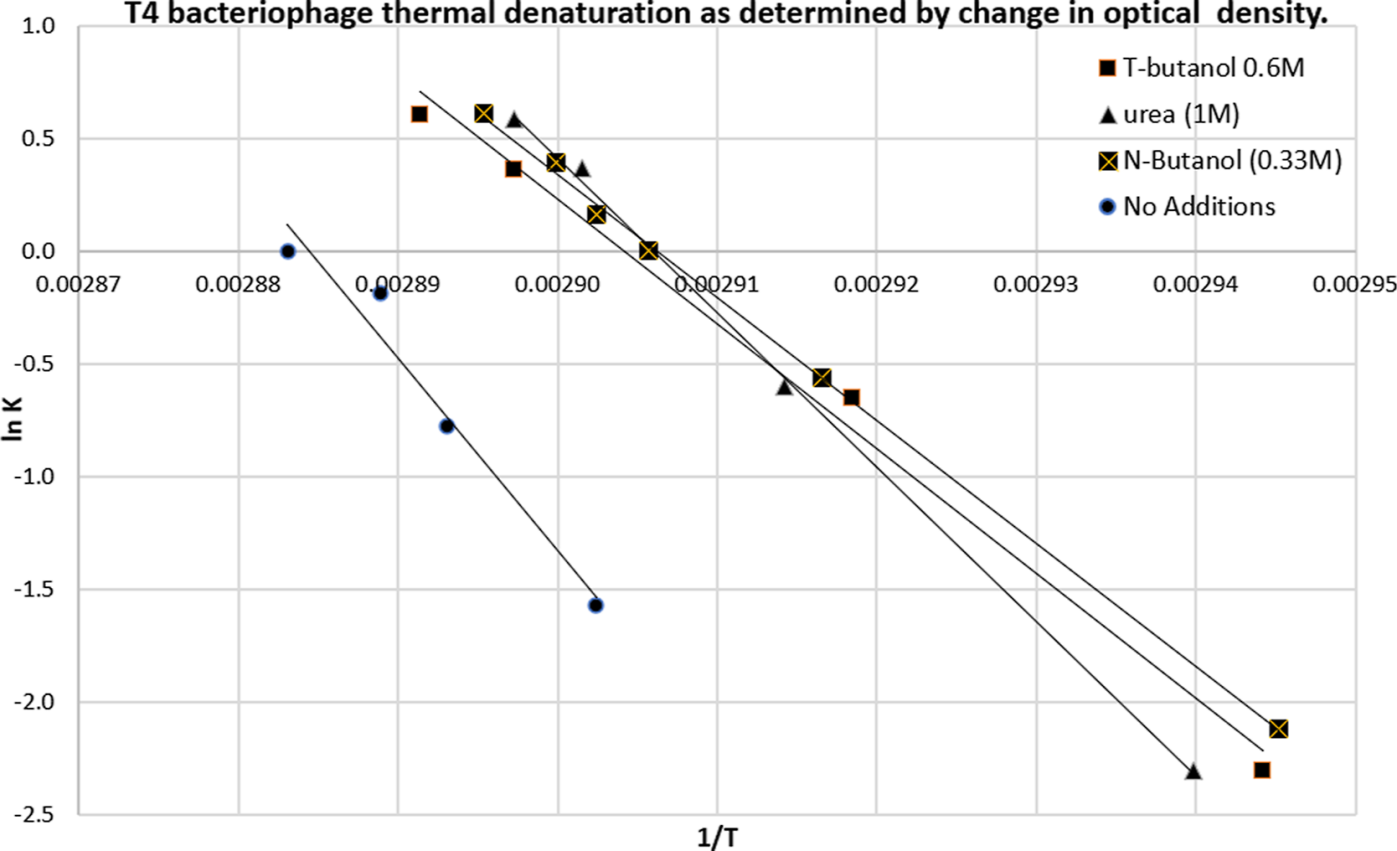
Thermal DNA stability as determined by change in optical density. Denaturation has been performed in the presence and absence of Urea, *N*-butanol, and *T*-butanol in Tris/EDTA buffer. Recalculated from the ACS publication ref 53 where *T* is in Kelvin.

Table 6 presents the enthalpy values for thermal denaturation calculated from the data of Figures 3 and 4. This table also contains the total enthalpy data for chemical DNA denaturation in solutions identical to those used for the thermal denaturation experiments.

The total enthalpies for chemical denaturation have been calculated using eq 34 according to experimental data of^53^ and Hansen parameters recalculated for the temperature of the experiment.

Experiments for chemical denaturation and measurements have been conducted at constant temperatures.^53^

These data show that the enthalpy for thermal denaturation is positive (endothermic process), and the enthalpy for chemical denaturation is negative (exothermic process). Also, the absolute enthalpy values for DNA chemical denaturation are significantly lower than for the thermal denaturation process.

For thermal and chemical denaturation, there are differences in *T*_m_. In the presence of urea or alcohol, the melting temperature for DNA thermal denaturation is 7 K to 8 K lower than for chemical denaturation in the same system (Table 5). This confirms the conclusion that there is a difference in the mechanism of reaching 50% DNA denaturation in this process’s thermal and chemical types.

**Table 5:** Comparison of Thermal and Chemical Denaturation of T4 Bacteriophage DNA in the Presence and Absence of Denaturants

additives^a^ to Tris + EDTA buffer	*M* for addatives^a^ mol·L^–1^	thermal denaturation process		enthalpy^c^ in chemical	difference
		*T*_m_ (K)	enthalpy^b^ (J·mol^–1^)	denaturation process at 346 K (J·mol^–1^)	(*T*_m_) chemical – (*T*_m_) thermal (K)
none	0	346.85	6,76,649	NA	NA
*n*-butanol	0.33	337.84	4,55,865	(43,980)	8.31
*t*-butanol	0.6	339.56	4,57,928	(44,058)	6.59
urea	1	337.84	5,64,285	(44,829)	8.31

a Value M is the concentration of denaturant required to give 50% denaturation of T4 bacteriophage DNA at 346 *K* in TRIS (0.05 M) EDTA (0.001 M) buffer. M values adapted from ref 53.

b The enthalpy calculated from the slopes on ln *K* = *f*(1/*T*) plots on Figures 3 and 4 multiplied by the universal gas constant.

c The total enthalpy calculated at *T*_m_ = 346 *K* using eq 34 with experimental data for *V*_f_ and Hansen parameters.

Table 6 summarizes the major differences between thermal and chemical types of DNA denaturation.

**Table 6:** Differences between the Thermal and Chemical DNA Denaturation Processes

thermal denaturation (A)	chemical denaturation (B)	notes
the thermal denaturation of DNA is the endothermic process where the enthalpy is positive.	the chemical denaturation of DNA is the exothermic process where the enthalpy is negative.	(A) see ln *K* vs 1/*T* slopes, Figures 3 and 4. (B) see the theory presented in this article with experimental verifications.
the Hildebrand equation does not apply to endothermal processes like thermal DNA denaturation.	the modified Hildebrand equation applies to exothermal processes such as chemical DNA denaturation.	see discussion in the section “DNA Denaturation in One Component Liquid Systems” related to expressions 4 and 27.
the value of enthalpy is significant: 450 – 650 kJ*mol^–1^ for T4 bacteriophage DNA	the value of enthalpy is small: (42–44) kJ*mol^–1^ for T4 bacteriophage DNA	see Table 5 for A and B sections.
the melting temperature for thermal denaturation is lower than chemical: 7 – 8 K for T4 bacteriophage DNA.	the melting temperature for chemical denaturation is higher than thermal: 7 – 8 K for T4 bacteriophage DNA	see Table 5 for A and B sections.
hydrogen bonding/forces have only entropic, not enthalpic origin, and these forces are responsible for approximately 40% of Gibbs free energy of DNA denaturation.^42^	hydrogen bonding has mainly enthalpic origin (67% of total enthalpy responsible for DNA denaturation). Disruption of hydrogen bonds is the dominant factor for denaturation	(A) see introduction for refs 42, 59, 60 and 101 (B) see Tables 2 and 4.
dispersion (apolar, van der Waals) forces are the main portion of denaturation enthalpy. They provide around 60% of Gibbs free energy for DNA denaturation.^42^	the dispersion component of enthalpy has a small influence (6–16%) on DNA denaturation.	(A) see introduction for refs 42, 59, 60 and 101 (B) See Tables 2 and 4.

The chemical denaturation process (at constant temperature) involves the replacement of initial bonds that kept DNA in double-stranded form, with the formation of new “DNA + denaturant” bonds with a higher energy of interaction, leading to the separation of DNA strands with the formation of the random coils or single-stranded state. The enthalpy of the first stage of chemical denaturation (breaking the bonds inside DS DNA) is Δ*H*_endo_. The enthalpy of the second stage of chemical denaturation (formation of new bonds with components of the surrounding solution) is Δ*H*_exo_. This process occurs spontaneously because the energy of interaction between active sites of DNA and components of the surrounding solution (including possible renaturation) should be higher or at least equal to the energy of initial inter-DNA bonding involved in the denaturation process or Δ*H*_exo_ ≥ Δ*H*_endo_ (See eq 21). Thus, chemical denaturation is an exothermic process, negatively affecting the net enthalpy.

The thermal denaturation process involves breaking DNA bonds with dominant van der Waals forces of an enthalpic nature.^42^ We have the enthalpy-dominated hydrogen bond limiting denaturation in the case of DNA chemical denaturation at experimental conditions of authors.^53^ Changes in DNA type, length, and conditions of the experiment might lead to energetically different limiting steps in the chemical denaturation process related to the increased role of dispersion forces. This is the second possible explanation for the differences in Δ*H* values for chemical and thermal processes.

## 6 Conclusions and Future Directions

### 6.1 Conclusions

1. We have developed a theory describing the chemical denaturation of DNA for low and medium denaturation degrees, including but not limited to 50% denaturation, as a reversible first-order reaction. The theory had no adjustable parameters; all parameters were known from independent data, and the theory was supported experimentally. This theory, based on experimental data, shows the degree of influence of hydrogen bonding, dispersion, polar forces, proton donor/acceptor ratio, dipole induction, orientation parameter, and electrostatic interaction during DNA denaturation. The theory links the degree of DNA denaturation with the concentration of denaturants, Hildebrand, Hansen, KSE cohesion parameters, and temperature.
2. Thermal and chemical DNA denaturation have different thermodynamic parameters. The enthalpy for thermal denaturation is positive (endothermic process), and the enthalpy of DNA melting for chemical denaturation is negative (exothermic process). Experimental results show that the absolute enthalpy values for DNA chemical denaturation are significantly lower than those in the thermal denaturation process. The melting temperature for DNA thermal denaturation is lower than for chemical denaturation in the same system, indicating that the mechanism to reach 50% DNA denaturation thermally and chemically is different.
3. Analysis of the chemical DNA denaturation process using Hansen-fractional three-component parameters shows that hydrogen bonding is the most significant part of the enthalpy of chemical denaturation for the DNA of the T4 bacteriophage. The experimental data for the five fractional cohesion parameters show that the proton-donor effect is the dominant mechanism in hydrogen-bonding changes during DNA denaturation. The influence of this effect is two times greater than that of the proton-acceptor effect. Another essential factor for DNA denaturation is the orientation component, which is part of the polar cohesion parameter. We also suggested that the total cohesion parameter measured at 50% DNA chemical denaturation represents the electrostatic (repulsion) forces that maintain the DNA helix.
4. Theoretical and experimental results show that the Hildebrand, Hansen, and KSE equations are applicable and instrumental in studying the chemical denaturation of DNA.
5. We have developed a method to reveal forces that maintain the integrity of the dsDNA helix under the thermodynamically proven indication that the distributions of the fractional cohesion parameters inside the DNA at its melting temperature are identical to those of the fractional cohesion parameters for the components in the solution. The method enables the calculation of inaccessible properties in polyelectrolytes from knowledge of other known or easily measurable properties of the surrounding solution. This method is important because it allows for estimating the degree of influence of electrostatic repulsion and different attraction forces on DNA during its chemical denaturation. This method can be suitable for selecting DNA (or other systems with controllable denaturation) targeted for new applications and denaturants suitable for effective denaturation.

### 6.2 Limitations and Future Directions

#### 6.2.1 Limitations

The limitation of the theory is the assumption of the additivity principle for physicochemical parameters of solution components surrounding polyelectrolyte molecules. It means that relatively simple solvents with low polarity should be selected to study the denaturation of DNA. All 31 solvents studied in the present article support the principle of additivity. However, we expect that extending this selection to more polar systems will result in a deviation from this principle. The additivity principle does not apply to cases where salts are present in solution.

#### 6.2.2 Future Directions

The additivity principle, on which our theory is based, does not apply to salt. A potentially significant future direction for our work involves determining how to expand the present model to account for the salt effect on polyelectrolyte denaturation.

The applicability of our theory across various DNA sequences and solvent types has not been thoroughly studied and warrants further investigation.

Some other future directions are.

- Extending the model to RNA and other nucleic acids using controllable denaturation as a tool. Denaturants selected for this purpose should have known values of fractional cohesion parameters. An example of RNA controllable chemical denaturation is the isothermal titration of RNA with a denaturant.^102^ The mixed chemical-thermal denaturation (see example in^103^) can be used as a control.
- The method may apply to the study of protein unfolding. The application of the method requires the selection of a controllable point, similar to the melting temperature for DNA denaturation. This point should detect the impact that a denaturant has during the transition between different structural assemblies of proteins (amyloid-like, intrinsically disordered, and globular classes) or changes within one class of proteins. Some examples of such points are shown in.^104,105^

- Estimating the Hansen and/or KSE fractional cohesion parameters for new compositions or systems of interest, and then applying our method for these new processes or product development.

For example, estimation of such cohesion parameters for supramolecular assemblies using cyanuric acid modified with a) alcohols and b) alkylamines, which have different aliphatic chain lengths, will open the possibility to understand the mechanism/role of different intermolecular forces during the formation of DNA-based or RNA-based supramolecular polymers.

Lachance-Brais, Sleiman et al.^100^ present dependencies of assembled fractions (degree of incorporation of modified cyanuric acid into DNA) versus the concentrations of modified cyanuric acid derivatives in the system. If our theory (that has only been supported for the DNA denaturation process) is applicable to this polymerization process, then this will enable the calculation of the specific intermolecular forces in the supramolecular assemblies and their change with the change in the chemical structure of the additive. This calculation is possible since the surrounding media’s cohesion parameters at 50% of the polymer’s assembly completion will be equal to those in the half a ssembled supramolecular polymer. For this point, the intermolecular forces acting during the copolymerization process can be revealed as soon as the fractional cohesion parameters for the modified cyanuric acids are available. Such knowledge will open the way to further polymer modification.

- Expand (create) the types of fractional cohesive energy parameters to include/reflect the unique formation characteristics of RNA, protein, and supramolecular assembly (and, if possible, ionic interactions that, for example, include RNA-cation interactions and salt bridge formation).
